# Effect of *in utero *exposure to diethylstilbestrol on lumbar and femoral bone, articular cartilage, and the intervertebral disc in male and female adult mice progeny with and without swimming exercise

**DOI:** 10.1186/ar3696

**Published:** 2012-01-23

**Authors:** Sora Al Rowas, Rami Haddad, Rahul Gawri, Abdul Aziz Al Ma'awi, Lorraine E Chalifour, John Antoniou, Fackson Mwale

**Affiliations:** 1 Lady Davis Institute for Medical Research, Sir Mortimer B. Davis-Jewish General Hospital, 3755 Chemin Cote Ste Catherine, Montréal, Quebec H3T 1E2, Canada; 2 McGill University Department of Surgery (Division of Surgical Research), Montreal General Hospital, 1650 Cedar Avenue, Room C9-160, Montreal, Quebec H3G 1A4, Canada; 3 Division of Experimental Medicine, McGill University, Room 101, Lady Meredith House, 1110 Pine, Avenue West, Montreal, Quebec H3A 1A3, Canada; 4 Bank of Montreal Research Center for the Study of Heart Disease in Women, Lady Davis Institute for Medical Research, Sir Mortimer B. Davis-Jewish General Hospital, 3755 Chemin Cote Ste Catherine, Montréal, Quebec H3T 1E2, Canada

## Abstract

**Introduction:**

Developmental exposure to estrogens has been shown to affect the musculoskeletal system. Furthermore, recent studies have shown that environmental exposure to estrogen-like compounds is much higher than originally anticipated. The aim of this study was to determine the effects of diethylstilbestrol (DES), a well-known estrogen agonist, on articular cartilage, intervertebral disc (IVD), and bone phenotype.

**Methods:**

C57Bl/6 pregnant mice were dosed orally with vehicle (peanut oil) or 0.1, 1.0, and 10 μg/kg/day of DES on gestational days 11 to 14. Male and female pups were allowed to mature without further treatment until 3 months of age, when swim and sedentary groups were formed. After euthanasia, bone mineral density (BMD), bone mineral content (BMC), bone area (BA), and trabecular bone area (TBA) of the lumbar vertebrae and femur were measured by using a PIXImus Bone Densitometer System. Intervertebral disc proteoglycan was measured with the DMMB assay. Histologic analysis of proteoglycan for IVD and articular cartilage was performed with safranin O staining, and degeneration parameters were scored.

**Results:**

The lumbar BMC was significantly increased in female swimmers at both the highest and lowest dose of DES, whereas the femoral BMC was increased only at the highest. The males, conversely, showed a decreased BMC at the highest dose of DES for both lumbar and femoral bone. The female swim group had an increased BA at the highest dose of DES, whereas the male counterpart showed a decreased BA for femoral bone. The TBA showed a similar pattern. Proteoglycan analysis of lumbar IVDs showed a decrease at the lowest doses but a significant increase at the highest doses for both males and females. Histologic examination showed morphologic changes of the IVD and articular cartilage for all doses of DES.

**Conclusions:**

DES significantly affected the musculoskeletal system of adult mice. Results suggest that environmental estrogen contaminants can have a detrimental effect on the developmental lumbar bone growth and mineralization in mice. Further studies measuring the impact of environmental estrogen mimics, such as bisphenol A, are then warranted.

## Introduction

Diethylstilbestrol (DES) is an estrogen agonist that was widely used between 1947 and 1975 to prevent miscarriages and to suppress postpartum lactation [1]. A large number of pregnant women were exposed to DES in North America, Europe, and Australia. The National Institutes of Health estimated that 5-10 million women were exposed during pregnancy [2]. Its use in agriculture was finally discontinued in 1979 [3]. The use of DES was discontinued because of major adverse effects on children born to DES-exposed mothers whose side effects included, but were not limited to, clear-cell vaginal adenocarcinoma in DES daughters, reproductive organ defects in both DES sons and daughters, and various cancers [4].^.^

Evidence suggests that *in utero *exposure to DES can induce epigenetic changes that affect the third generation [5]. The phenotype associated with these epigenetic changes is not yet characterized. The youngest sons and daughters that were exposed to DES (discontinued in 1971 in the United States) still have 15 reproductive years left [6]. Understanding the effects of DES on various estrogen-responsive tissues thus continues to be important today [4].

Estrogen affects articular cartilage and intervertebral disc (IVD) turnover [7-10]. Its treatment in postmenopausal women led to an increase in IVD height when compared with that of untreated postmenopausal women [8]. It was found to increase the synthesis of proteoglycans in articular chondrocytes from surgically menopausal monkeys [9] and to suppress collagen breakdown in ovariectomized rats [10]. These results indicate that estrogen can have a direct effect on adult IVD and articular cartilage. Because DES is a potent estrogen agonist [7], it would be reasonable to assume that it could have an effect on the IVD, as well as articular cartilage. Exposure of newborn mice to DES induces permanent changes in skeletal tissue in adulthood, such as shorter femurs and increase in the amount of bone in the femurs and vertebrae [11,12], suggesting that physiological exposure to estrogens in childhood might be one of the key factors in determining the final peak bone density in adulthood. However, little is known about fetal exposure to DES on articular cartilage and the IVD in male and female adult progeny.

Pregnant women were typically started on a low-dose regimen of DES, which was escalated every 2 weeks to a maximum dose of 0.15 g/day until the end of the pregnancy [8]. However, animal studies have shown that even a transient exposure to DES can affect bone, both during gestation and postnatally. A dose as low as 2 μg/pup/day was previously used between postnatal days 1 and 5 to affect femoral and vertebral bone in females [9]. Another study used doses ranging from 0.1 μg/kg/day to 100 μg/kg/day between days 9 and 16 of gestation to examine the effect of DES on female femoral and vertebral bone [10].

Environmental exposure to estrogen agonists like bisphenol A have recently been described to be more prevalent in urban areas [13]. It has also been suggested that findings from the study of one estrogen agonist can be extrapolated onto another [6]. In an aging population, musculoskeletal health becomes ever more important, as identifying risk factors for early fractures and/or degenerative processes can help prevent significant morbidity in otherwise vital members of society. This study aims to examine the effects of a brief *in utero *exposure to three different doses of DES on the musculoskeletal system of adult progeny, which would shed light on the possible increased risk of fracture in the sons and daughters of mothers exposed to DES during gestation, as well as in their children. Furthermore, this new-found environmental exposure to estrogen agonists makes it important to understand the full effects of such estrogen agonists.

## Materials and methods

All experiments were performed according to the guidelines of the Canadian Council of Animal Care and Animal Care Committee of the Lady Davis Institute for Medical Research, consistent with those of the National Institutes of Health.

### Animal manipulation

Pregnant C57/bl mice were injected with either vehicle (peanut oil) or one of three doses of DES (Sigma Aldrich, Oakville, Ontario) (0.1, 1.0, and 10.0 μg/kg/day) at 11 to 14 days of gestation. As described earlier, these doses were previously shown to affect bone mass and mineralization when used during gestation. A minimum of three dams was set for each group. Pups were then allowed to grow to adulthood without further intervention until 3 months of age. At this point, mice were randomized into two groups; one with a once daily swimming regimen, which started at 5 minutes and was escalated to a maximum of 1 hour within a week. Animals had 4 weeks of total swim time before being euthanized. Mice from the second group were left to their normal activity level and considered sedentary (see Table 1). All animals were euthanized at exactly 4 months of age, weighed, and frozen at -20°C.

**Table 1:** Number of pups in each group

	Female swim^a^				Female sedentary^b^				Male swim^a^				Male sedentary^b^			
	Control^c^	0.1 DES^c^	1.0 DES	10.0 DES	Control	0.1 DES	1.0 DES	10.0 DES	Control	0.1 DES	1.0 DES	10.0 DES	Control	0.1 DES	1.0 DES	10.0 DES
No. of pups	8	6	8	8	9	7	5	11	6	8	5	7	11	8	7	9

**^a^**Female or male mice were put on a once-daily swimming regimen, which started as 5 minutes and was escalated to a maximum of 1 hour within 1 week. **^b^**Female or male mice were left to their normal activity level and considered sedentary. **^c^**Pregnant C57/bl mice were injected with either vehicle (peanut oil) or one of three doses of DES (0.1, 1.0, and 10.0 μg/kg/day) at 11 to 14 days of gestation.

### Bone densitometry analysis

Lumbar and femoral bones were evaluated by using bone densitometry to determine whether *in utero *DES exposure caused structural changes and whether this effect was gender specific. Swim and sedentary groups were compared to determine whether exercise potentiates the effect of DES on the musculoskeletal system. The bone mineral density (BMD), generally measured to determine fracture risk clinically, was evaluated as a surrogate marker of altered morphology [11]. The lumbar and femoral regions of each intact mouse were scanned by using the Piximus Bone Densitometry system, as previously described [11]. BMD, bone mineral content (BMC), bone area (BA), and trabecular bone area (TBA) were all measured for both lumbar and femoral bones. This was performed for all mice before dissection for other investigations. The BA was evaluated to determine whether the increase in mineral deposits affected bone growth, whereas the TBA was examined to determine bone quality.

### Micro-CT

Dissected and fixed samples of lumbar spine and femurs were placed in an imaging chamber. Percentage bone volume, trabecular thickness, trabecular number, trabecular separation, and distance between lumbar vertebrae were determined with a desktop Micro-CT scanner (SkyScan-1072; Skyscan, Aartselaar, Belgium) fitted with a sealed microfocus x-ray tube with a spot size less than 8 μm that operates at 20 to 100 kV/0 to 250 mA. The special x-ray CCD camera is based on high resolution (1,024 × 1,024 pixels) cooled CCD-sensor. The following settings were chosen: X-ray source, 45 kV, and at 222 μA resolution: 9.38 μm, rotation step: 0.9 degrees, exposure time: 7,500 milliseconds. The resolution was chosen because it was found to be optimal for measurements. Reconstruction was performed by using NRecon (SkyScan). CT-Analyser and 3D Creator (SkyScan) were used for quantitative analyses and 3D rendering, respectively.

### Histologic examination

The lumbar region and knee joint (mid femur to mid tibia) were dissected from two animals per group for histologic evaluation, fixed in 10% formalin for 18 hours, decalcified in formic acid for a maximum of 48 hours, dehydrated in graded solutions of ethanol in water, infiltrated with xylenes, and finally paraffin embedded, as previously described [12,14,15]. Sections of 4 μm were cut with a microtome and placed on glass slides. After deparaffinization, they were stained with 0.1% safranin O and counter stained with 0.02% Fast Green. All slides were scanned by using the *NDP view *software (Hamamatsu Corp., Hamamatsu, Japan). Several parameters previously identified as correlated with articular cartilage degeneration were evaluated and are described in Table 2[16-18]. Selected parameters were chosen from the Boos grading scheme to score IVD degeneration, shown in Table 3[19]. Quantitative histologic evaluation was not performed because of the small number of animals, but the available data are included for completion.

**Table 2:** Articular cartilage histologic evaluation criteria

Criteria for histologic evaluation of articular cartilage	
Safranin O-fast green staining	
0	No loss
1	Mild loss
2	Moderate loss
3	Severe loss
Chondrocyte loss	
0	No decrease in cells
1	Minimal decrease in cells
2	Moderate decrease in cells
3	Marked decrease in cells
4	Very extensive decrease in cells
Structure	
0	Normal
1	Surface irregularities
2	One to three superficial clefts
3	More than three superficial clefts
4	One to three clefts extending into the middle zone
5	More than three clefts extending into the middle zone
6	One to three clefts extending into the deep zone
7	More than three clefts extending into the deep zone
8	Clefts extending to calcified cartilage
Chondrocyte clones	
0	Normal
1	Minimal (≤4)
2	Moderate (> 4 but ≤8)
3	Marked (> 8)
Tidemark integrity	
0	Intact
1	Crossed by blood vessels
Tidemark duplication	
0	No change
1	Duplication of tidemark
2	No visible tidemark remaining
Subchondral bone thickness	
0	Subchondral bone thickness less than thickness of cartilage
1	Subchondral bone thickness equal to thickness of cartilage
2	Subchondral bone thickness more than thickness of cartilage

**Table 3:** Histologic evaluation parameters for the intervertebral disc

Parameters	Grade	Description
Clefts in the NP	0	Absent
	1	Rarely present
	2	Intermediately present
	3	Abundantly present
Tears in AF	0	Absent
	1	Rarely present
	2	Intermediately present
	3	Abundantly present
Granular changes	0	Absent
	1	Rarely present
	2	Intermediately present
	3	Abundantly present
Mucous degeneration	0	Absent
	1	Rarely present
	2	Intermediately present
	3	Abundantly present
Cell clustering	0	No proliferation
	1	Mild
	2	Moderate
	3	Severe

### Proteoglycan content

Cervical to lumbar IVDs from three mice were isolated and digested overnight at 56°C with proteinase K (Sigma Aldrich, St. Louis, MO, USA). They were then vortexed and centrifuged for 30 minutes at 2,800 *g*. IVD proteoglycans in the supernatant were measured by using the DMMB assay [20]. Shark chondroitinase (Sigma Aldrich, Cambridge, MA, USA) was used for the standard curve. Standard curve was generated with concentrations ranging from 10 μg/ml to 50 μg/ml, and colorimetric reads were taken with a DU 640 spectrophotometer (Beckman, Carlsbad, CA) at 595 nm.

### Statistical analysis

All statistical analyses were done by using ANOVA and Fisher Least Significant Difference *Post Hoc *test with StatView Software version 4.0 (SAS Institute, Cary, NC). Assuming a comparison of means over the four dosage groups, a sample size of five animals per group would provide at least 80% power to detect a large effect size (*f *= 0.86) against a null value (this is, no difference in the means) at a critical test level of 5%. The effect size of 0.86 indicates that the standard deviation of the means is 86% as large as the standard deviation of the observations. No statistical analysis was performed for histology data because of the small sample size. Those results are included as a point of interest and to supplement the data obtained from imaging and proteoglycan analysis.

## Results

Table 1 shows the number of pups born to each group of dams. No significant differences were noted in terms of number of live births for each dosage of DES. Pups born to different mothers were treated equally, because the mothers were identical genetically and had no environmental differences other than the dose of DES given during gestation.

### Radiographic assessment

**Figure 1:**
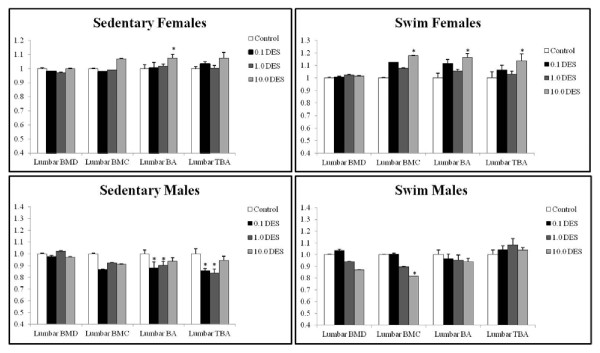
**Lumbar bone densitometry with DES treatment**. Bone mineral density (BMD), bone mineral content (BMC), bone area (BA), and trabecular bone area (TBA) were measured in animals at death with a PIXImus Bone Densitometer System and analyzed by using a Windows 98-based software. Ratio of mean to control is plotted for each parameter plus the standard deviation. Significance was calculated in comparison to the vehicles by using ANOVA and is reported when *P *< 0.05 versus peanut oil controls.

Several doses of DES significantly affected lumbar BMC, BA, and TBA (Figure 1). The lumbar BMD was not significantly affected at any dose of DES. The BMC was measured to determine whether the lack of effect on BMD meant that no changes in the calcium deposits had occurred. Surprisingly, the lumbar BMC was significantly increased at 10.0 μg/kg/day (18%; *P *= 0.0176) for the female swim group. However, it was significantly decreased (8%; *P *= 0.0498) at 10.0 μg/kg/day of DES in the male swim group (Figure 1).

The lumbar BA showed a significant increase at 10.0 μg/kg/day (7%; *P *= 0.0341) in the female sedentary group and at both 0.1 (12%; *P *= 0219) and 10.0 μg/kg/day (16%; *P *= 0.0006) in the female swim group. Lumbar BA in the male sedentary group showed a significant decrease at 0.1 (2%; *P *= 0.0045) and at 1.0 μg/kg/day (2%; *P *= 0.0218). The male swim group showed no significant differences in lumbar BA, although a trend of decreased lumbar BA compared with control was seen; this did not reach statistical significance.

The lumbar TBA was not significantly affected in the female sedentary group. The lumbar TBA in the female swim group significantly increased at 10.0 μg/kg/day (14%; *P *= 0.0178). The male sedentary group showed a markedly significant decrease in lumbar TBA at 0.1 and 1.0 μg/kg/day (14% and 15%; *P *< 0.0001 for both), but no significant difference was seen at 10.0 μg/kg/day. No significant effect was seen in the male swim group lumbar TBA (Figure 1).

**Figure 2:**
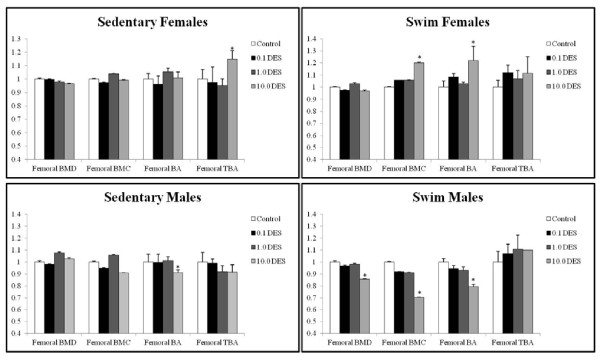
**Femoral bone densitometry with DES treatment**. Bone mineral density (BMD), bone mineral content (BMC), bone area (BA), and trabecular bone area (TBA) were measured in animals at death with a PIXImus Bone Densitometer System and analyzed by using a Windows 98-based software. Ratio of mean to control is plotted for each parameter plus the standard deviation. Significance was calculated in comparison to the vehicles by using ANOVA and is reported when *P *< 0.05 versus peanut oil controls.

Fetal exposure to several doses of DES significantly affected femoral BMD, BMC, BA, and TBA (Figure 2). Femoral BMD was calculated to see whether the effect of *in utero *exposure to DES had regional specificity. Femoral BMD was significantly decreased in the male swim group at 10.0 μg/kg/day (15%; *P *= 0.0055). Otherwise, no significant effect on femoral BMD was seen in the other groups. The femoral BMC was measured to evaluate whether the effect on BMD was consistent with decreased mineral deposits in femoral bone. No effect on femoral BMC was seen in sedentary females (Figure 2). The swim female femoral BMC showed a significant increase at 10.0 μg/kg/day (20%; *P *= 0.0369). No effect was seen on femoral BMC in sedentary males. The swim male femoral BMC showed a markedly significant decrease at the highest dose of DES (31%; *P *< 0.0001).

The female swim femoral BA significantly increased at 10.0 μg/kg/day (22%; *P *= 0.0361). The female sedentary group femoral BA was not significantly affected by DES. Both male swim and sedentary groups were affected at the highest dose of DES exposure and showed significant decreases in femoral BA (swim, 9%; *P *< 0.0001; sedentary, 21%; *P *< 0.0001). At 0.1 and 1.0 μg/kg/day of DES, males showed a decrease in femoral BA that did not reach statistical significance.

The femoral TBA was not significantly affected in the female swim group. The female sedentary group showed a significant increase at 10.0 μg/kg/day (14%; *P *= 0.0186). Otherwise, no significant differences were seen in femoral TBA. The male sedentary group showed a 10% decrease in femoral TBA at all doses of DES, although it was not statistically significant (Figure 2).

The goal of micro-CT was to assess further the results obtained with bone densitometry; thus only the control and the highest dose of DES were analyzed for sedentary females. Fetal exposure to DES had significant effects on lumbar bone in terms of the percentage of bone volume, trabecular separation, trabecular number, and distance between lumbars (Table 4). The percentage bone volume (PBV) is a measure of the quantity of cortical bone with respect to total bone volume [21]. PBV significantly decreased at 0.1 μg/kg/day (8%; *P *= 0.014429) and increased at 10.0 μg/kg/day (27%; *P *= 0.002076). The trabecular separation, thickness, and number are measures of the quality of cancellous bone [22] and were evaluated to correlate the trabecular bone-area measures to densitometry. The trabecular separation of lumbar bone only significantly decreased at 10.0 μg/kg/day (24%; *P *= 0.00235), but a 15% increase was noted at 0.1 μg/kg/day, which was not statistically significant. The trabecular number in lumbar bone was significantly increased at both the lowest and highest dose (24%; *P *= 0.029284; and 28%; *P *= 0.001317). The distance between lumbars is a surrogate measure for IVD height [23]. It was significantly decreased at 1.0 μg/kg/day (30%; *P *= 0.00513). The trabecular thickness of lumbar bone was not significantly affected by DES at any dose. This increased trabecular number, coupled with a decreased trabecular separation, correlates with the noted increase in TBA and BMC at these doses of DES. These characteristics are consistent with more-fragile bone.

**Table 4:** Sedentary female lumbar micro-CT

Sedentary female		Percentage bone volume (%BV/TV)	Standard deviation	*P *value
	**Control**	13.19317	1.42201	
μg/kg/day DES	**0.1**	12.18233	0.881048	**0.014429**
	**1.0**	13.29047	0.259911	0.92804
	**10.0**	16.80974	1.612495	**0.002076**
		Trabecular separation (mm)		
μg/kg/day DES	Control	0.34907	0.045484	
	0.1	0.404715	0.010953	0.457184
	1.0	0.321765	0.016935	0.616302
	10.0	0.266997	0.020094	0.00235
		Trabecular thickness (mm)		
μg/kg/day DES	Control	0.075187	0.003084	
	0.1	0.077025	0.000601	0.455911
	1.0	0.078255	0.001237	0.236835
	10.0	0.074538	0.001627	0.658458
		Trabecular number (1/mm)		
μg/kg/day DES	Control	1.757235	0.200325	
	0.1	2.18268	0.016843	0.02928
	1.0	1.698535	0.03334	0.70857
	10.0	2.25342	0.189375	0.001317
		Distance between lumbars (μm)		
μg/kg/day DES	Control	314.5833	44.74319	
	0.1	262.5	33.78959	0.097763
	1.0	218.75	10.82532	0.00513
	10.0	297.875	69.96584	0.579692

Female sedentary femoral bone was not significantly affected by fetal exposure to DES, but some trends were noted (Table 5). The percentage bone volume showed an average increase of 45%, which was not statistically significant because of one outlier. If this is disregarded, then the difference is significant, with *P *= 0.02. The trabecular separation was decreased by 35% with a nonsignificant *P *= 0.07009. The trabecular thickness and trabecular number showed no significant changes at any dose.

**Table 5:** Sedentary female femoral micro-CT

Sedentary female		Percentage bone volume (%BV/TV)	Standard deviation	*P *value
μg/kg/day DES	Control	1.81240	2.285448	
	10.0	2.636213	0.612359	0.578992
		Trabecular separation (mm)		
μg/kg/day DES	Control	0.543937	0.137587	
	10.0	0.34779	0.015002	0.07009
		Trabecular thickness (mm)		
μg/kg/day DES	Control	0.05791	0.007864	
	10.0	0.054943	0.003897	0.589679
		Trabecular number (1/mm)		
μg/kg/day DES	Control	0.290977	0.340796	
	10.0	0.481133	0.114544	0.411454

### Histology

**Figure 3:**
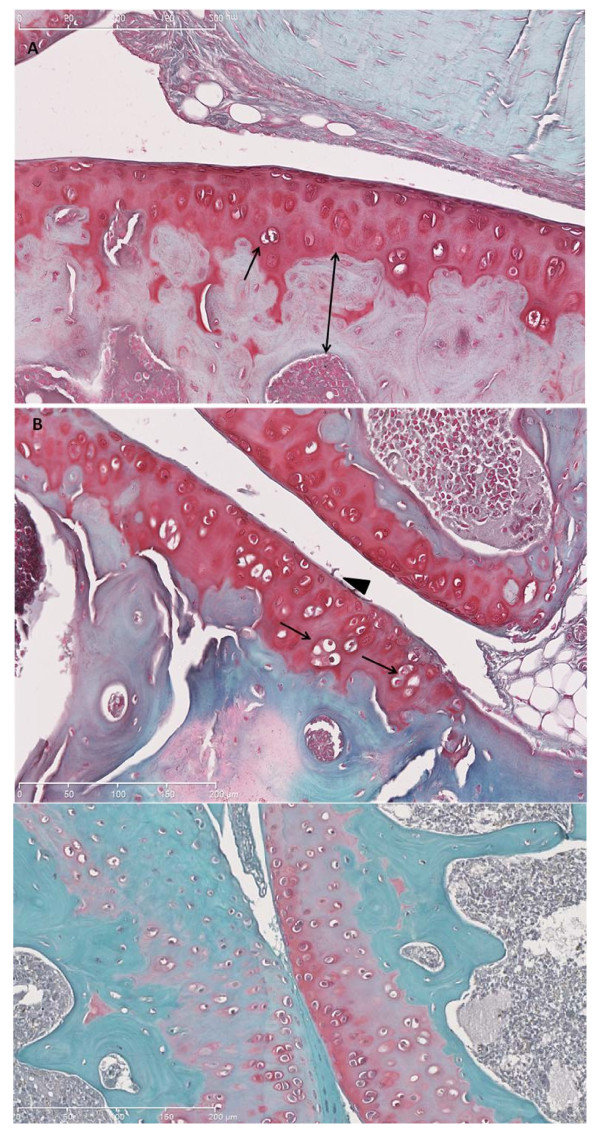
**Representative safranin O-fast green-stained histologic sections**. **(a) **Normal articular cartilage from female swim control. Single-headed arrow shows chondrocytes clones. Double-headed arrow demonstrates subchondral bone thickness. **(b) **Male swim 1.0 DES limb; note abundant chondrocyte clones (single-headed arrow) and fibrillation (arrowhead). **(c) **Male swim, 1.0 DES. Note paucity of staining, chondrocytes proliferation, and duplication of tide mark (white arrowhead).

Staining with safranin O of the IVD and cartilage, which detects the chondroitin sulfate chains of aggrecan to evaluate parameters, is described in Tables 2 and 3. Red safranin-O staining was present throughout the tissue (Figure 3).

**Figure 4:**
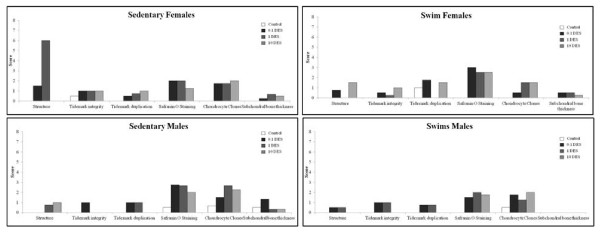
**Histologic scores for articular cartilage at 16 weeks**. Histologic parameters are compared between groups. Histologic analysis revealed that loss of proteoglycan, based on safranin O-fast green staining in all four groups at all doses of DES.

Loss of staining, chondrocytes clones, and some structural changes were most notable in articular cartilage from DES-exposed mice (Figure 3). Grossly, degenerative changes were noted in articular cartilage of DES-exposed animals at a higher rate than those in controls. Figure 4 shows articular cartilage scores for each parameter analyzed and demonstrates that scores were consistently higher in DES-exposed animals than in controls.

**Figure 5:**
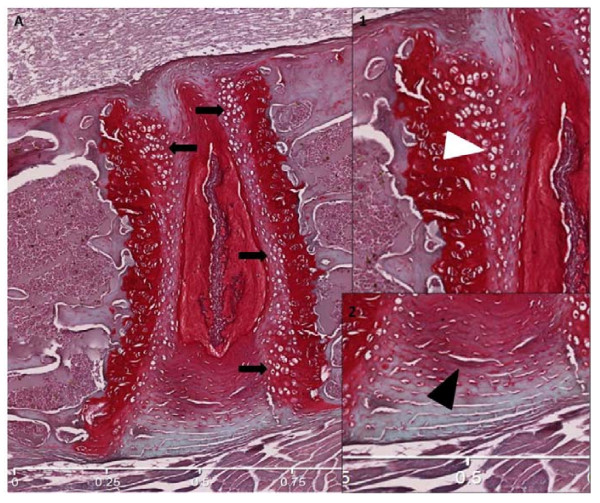
**Representative safranin O-fast green histologic sections**. **(a) **IVD from control female swim. Bold arrows show cellular proliferation. Inset 1: white arrowhead shows cellular clones. Inset 2: Black arrow shows AF fissuring grade 1. **(b) **IVD from 10.0 DES female swim group. White block arrow shows nuclear clefting. Inset 1: Granulation under the endplate.

**Figure 6:**
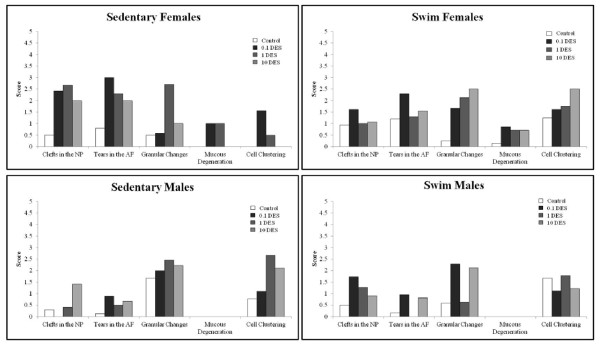
**Histologic scores of IVD at 16 weeks**. Various histologic parameters of degeneration showed higher scores in DES-exposed groups as compared with controls.

IVD showed increased granular changes, chondrocyte proliferation, and increased clefts and tears in both the AF and the NP in response to *in utero *DES exposure (Figure 5). Parameters associated with IVD degeneration were found to have higher scores in DES-exposed mice versus controls (Figure 6). Females exposed to DES seemed to display more difference from controls than did males exposed to DES, but more animals must be tested to verify this pattern.

### Proteoglycan

**Figure 7:**
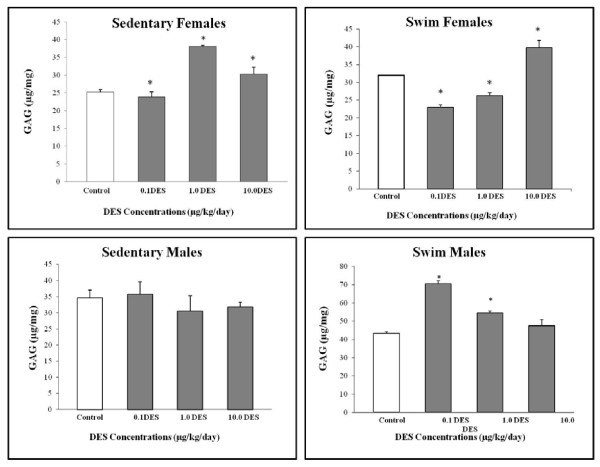
**Lumbar intervertebral disc proteoglycan analysis**. Whole intervertebral discs were analyzed with the DMMB assay. Significant differences in proteoglycan content were noted at all three doses of diethylstilbestrol (DES), as compared with control.

Proteoglycan content, predominantly aggrecan, was determined because it forms a major structural component of cartilage [24]. A higher proteoglycan content is a hallmark of healthy IVDs, as it signifies an improved ability to imbibe water, thus countering loads on the spine [25,26]. Lumbar IVDs showed significant changes in proteoglycan content at all doses of DES in females and at only certain doses in the male swim group (Figure 7). Proteoglycan content in the female swim group was significantly decreased compared with control at 0.1 μg/kg/day (28%; *P *= 0.0013) and 1.0 μg/kg/day (18%; *P *= 0.0072), whereas a significant increase was noted at 10.0 μg/kg/day (20%; *P *= 0.0024). The female sedentary group showed a significant increase in proteoglycan content only at the middle and highest doses (51%; *P *= 0.0005; and 20%; *P *= 0.0172). This group showed a decrease in the proteoglycan content at the lowest dose, which was not statistically significant. The male sedentary group showed no significant changes in proteoglycan content at any dose of DES. The male swim group, however, showed significant increases at 0.1 and 1.0 μg/kg/day of DES (62%; *P *< 0.0001; and 25%; *P *= 0.0063).

## Discussion

Estrogen-responsive organs are affected by estrogen imprinting well into adulthood [27]. These effects can depend on the duration of exposure during gestation as well as on the levels of estrogen [28,29]. Bone and articular cartilage have already been characterized as estrogen-responsive tissues [30,31]. More recent evidence suggests that the IVD contains estrogen receptors as well [32].

Developmental exposure to DES between 9 and 16 days of gestation was shown to increase both trabecular and cortical femoral bone mass in female offspring [10]. No similar studies evaluated the effect on either male offspring or the lumbar vertebrae. Although sexual dimorphism does exist, when it comes to the bone response to estrogen, estrogen-deficient males still have low bone mass [33]. Estrogen-receptor alpha (ERα) and beta (ERβ) are mainly responsible for the effects of estrogen on bone in both genders [34]. The involvement of estrogen receptor-related receptor (ERR) was also recently identified in the effects of estrogen on bone [35]. Diethylstilbestrol is known to have an agonistic effect on both ERα and ERR [35,36]. In light of these data, it would be expected that an increased BMD would be noted in both males and females, which would most likely be due to an increase in BMC.

The current study demonstrated that the BMC and TBA increased in adult females but decreased the overall bone size in males. This corresponds to more-fragile bone in females and a feminized phenotype in males because of the decreased bone size. The epigenetic changes induced by DES can thus be responsible for altering how bone responds to estrogen exposure [37,38]. Hypermethylation of histone 2 occurred in cell cultures exposed to DES and may play a part in the effect on bone [39]. The effects were similar in both lumbar and femoral bone, although lumbar bone was more sensitive to lower doses of DES. Clinically, this might imply vertebral fractures earlier in life, which can be associated with significant morbidity [40]. If other exercises that involve impact or high rates of load, such as running or jumping, as opposed to swimming, were evaluated, it is possible that lumbar and vertebral bone would have similar results. DES cohorts can thus potentially benefit from earlier screening with bone scans to detect those vulnerable to vertebral or hip fracture.

In this work, because the mothers were all epigenetic siblings and were treated exactly the same way during gestation (except for DES dose given), no reason exists to believe that pups born to different mothers have any reason to be different, except for the dose of DES to which they were exposed. All pups that received the same dose of DES during gestation were treated as being part of the same pool and randomized to swim or sedentary groups accordingly. The only difference between the sedentary and exercise groups was the exercise regimen. According to our results, it seems that exercise does potentiate the effects of DES. It could also be that DES affects how the musculoskeletal system responds to swimming. The fact that these differences were observed without weight-bearing exercise makes the results more striking. One of the limitations of the study was the small sample size. The sample size used in this study was determined based on the expense of data collection and expedience.

Humans were typically exposed to DES throughout gestation until birth [8]. The brief exposure in this study was chosen at a time of organogenesis; however, the overall dose of DES in this study is much less than the comparable dose used on pregnant women. Therefore, the present results are more likely to underestimate the effects of DES because the exposure was brief and at a lower cumulative dose. In this study, no dose-response relation was found. At first glance, this is not what one would expect. However, the lack of a dose response is actually typical for steroid hormones [38]. This is because estrogen receptors can have different downstream actions, depending on whether they were exposed to low or high doses of estrogen [41] This is why it is quite common to observe an effect at low and high doses of estrogen (or estrogen agonists) but not at intermediate doses.

DES is traditionally thought of as a pure estrogen agonist, but can actually have an antagonistic effect in some circumstances. It has an agonistic effect on ERα but an antagonistic effect on ERR-γ [42]. DES was most studied in terms of its effect on the reproductive system, and it was found to have a net agonist effect on these tissues (endometrium, breast, prostate) [33]. How a tissue responds to DES would largely be determined by the receptor distribution in that tissue. A prevalence of ERα in articular cartilage, for example, would lead us to suspect an agonistic effect. Furthermore, it should be noted that the effects observed in this study are due to "priming" with DES at a critical stage, which differs from exposing an adult animal to DES. This priming can change the receptor distribution in a particular tissue or in the downstream pathways that a particular receptor stimulates. These exact effects are not known at present and must be further elucidated.

Contradictory evidence exists about the effects of estrogen on articular cartilage [43]. Most studies, however, have concentrated on the effects of adult estrogen exposure on articular cartilage, which contains ERα [44]. Little is known about how estrogen imprinting with DES can affect articular cartilage. The present study found that even a brief 4-day fetal exposure to DES can increase markers of articular cartilage degeneration in adult mice. Safranin O staining demonstrated consistent decrease in proteoglycans for both males and females at all doses of DES. This suggests that *in utero *DES exposure can impair the ability of adult articular cartilage to maintain its proteoglycan content. Estrogen was shown to help maintain the integrity of articular cartilage in sheep and rats [45,46]. The detrimental effects seen here could be due to DES inhibiting the response of articular cartilage to estrogen in both adult males and females.

The intervertebral disc [IVD) was shown to express estrogen receptor β (ERβ) in the annulus fibrosus [32]. Diethylstilbestrol has an agonistic effect on ERβ, although it has a higher affinity for ERα [47,48]. These findings suggest that the IVD is an estrogen-responsive tissue, but no work has been done to study the effect of *in utero *exposure to DES on the IVD. The present study suggests that IVD in both male and female mice fetally exposed to DES are more susceptible to disc degeneration. Swimming ameliorated some of these effects but exaggerated others, suggesting different mechanisms of response to *in utero *exposure to DES. Swimming was studied as an alternative to weight-bearing exercise because it can have beneficial effects on bone turnover, strength, and structure [49,50]. More animals must be tested to corroborate that these degenerative changes are consistent.

Other than ERβ, no other estrogen receptors were detected in the IVD [51]. The altered proteoglycan content in DES mice can thus be a secondary effect. Sexual dimorphism was also noted in the IVD response to *in utero *DES exposure, replicating the pattern noted in bone and articular cartilage.

## Conclusions

*In utero *exposure to DES thus affected lumbar and femoral bone of both male and female adult progeny. Specifically, a higher BMC was noted in females, and a decreased overall bone size was noted in males. These findings correspond to more-fragile bone in females and a feminized phenotype in males. Degenerative changes were noted in the articular cartilage and IVD of relatively young adult mice. DES cohorts could thus benefit from earlier screening for signs of osteopenia or degenerative changes of their bone. ERα knockouts would be a good next step in elucidating the mechanisms of DES action on bone, articular cartilage and the IVD, as well as studies in older mice. Studies with large sample sizes are also warranted to corroborate the findings noted in articular cartilage and IVD.

## Abbreviations

AF: annulus fibrosus; BA: bone area; BMC: bone mineral content; BMD: bone mineral density; CT: computed tomography; DES: diethylstilbestrol; ER: estrogen receptor; GAG: proteoglycan; IVD: intervertebral disc; NP: nucleus pulposus; TBA: trabecular bone area.

## Competing interests

The authors declare that they have no competing interests.

## Authors' contributions

SAR performed all experiments that led to production of figures, analyzed the data, and wrote the article. RH performed all animal handling up to and including the point of euthanasia. RG helped address the reviewer's comments and modified the figures accordingly, in addition to contributing expertise and important feedback during the conduction of experiments. He was also instrumental in trouble shooting while experiments were ongoing. AAAM helped with the dissection and with the editing of the figures and the article. LC designed the experimental model for administration of diethylstilbestrol and for dividing animals into swim and sedentary groups. JA provided feedback and suggestions during laboratory meetings, helped with experimental design, and approved the final version of the article. FM designed experiments to determine effect of diethylstilbestrol on the musculoskeletal system, edited several drafts of the article, and had the final say as to which figures would be included. All authors have read the final manuscript and approved it for final publication.
